# [Retracted] MicroRNA‑9 limits hepatic fibrosis by suppressing the activation and proliferation of hepatic stellate cells by directly targeting MRP1/ABCC1

**DOI:** 10.3892/or.2023.8590

**Published:** 2023-06-19

**Authors:** Jie Sun, Huanying Zhang, Liying Li, Lianfeng Yu, Lifang Fu

Oncol Rep 37: 1698–1706, 2017; DOI: 10.3892/or.2017.5382

Following the publication of this article, a concerned reader drew to our attention that in Fig. 5C on p. 1704, showing histological images of mouse livers stained with H&E, unexpected areas of similarity were identified in terms of the staining patterns revealed within the data panels themselves.

After having conducted an internal investigation, the Editor of *Oncology Reports* has reached the conclusion that the overlapping portions of data shown in this figure were unlikely to have arisen by coincidence. Therefore, on the grounds of a lack of confidence in the integrity of these data, the Editor has decided that the article should be retracted from the publication. The authors were asked for an explanation to account for these concerns, but the Editorial Office did not receive any reply. The Editor apologizes to the readership for any inconvenience caused, and thanks the interested reader for drawing this matter to our attention.

